# Experiences of Patients With Borderline Personality Disorder With Imagery Rescripting in the Context of Schema Therapy—A Qualitative Study

**DOI:** 10.3389/fpsyt.2020.550833

**Published:** 2020-12-03

**Authors:** Anja Schaich, Diana Braakmann, Anja Richter, Clara Meine, Nele Assmann, Sandra Köhne, Arnoud Arntz, Ulrich Schweiger, Eva Fassbinder

**Affiliations:** ^1^Department of Psychiatry and Psychotherapy, University of Lübeck, Lübeck, Germany; ^2^Department of Clinical Psychology, University of Amsterdam, Amsterdam, Netherlands

**Keywords:** borderline personality disorder, imagery rescripting, schema therapy, qualitative research, perspective

## Abstract

Imagery Rescripting (IR) is a therapeutic technique that is used in a wide spectrum of therapeutic methods for various mental disorders. As an important component of Schema Therapy (ST), IR is frequently used in the treatment of patients with borderline personality disorder (BPD). However, little is known about how IR is experienced by individuals with BPD. The aim of this study was to explore BPD patients' experiences with receiving IR. Qualitative data were collected through semi-structured interviews with 21 individuals (86% females) with a primary diagnosis of BPD who received IR within their ST treatment. Interview data were analyzed following the procedures of qualitative content analysis. Participants reported various effects of IR including initial high emotionality and exhaustion. Long-term effects included a better understanding of schemas and an improvement regarding emotion regulation and interpersonal relationships. Participants reported factors hindering the successful implementation of IR, such as external noise, stress, and a fast pace during IR. Facilitating factors included adequate time for debriefing, a transparent structure, and preparation of IR as well as the therapist providing safety. Implications of the findings for optimizing IR in clinical practice are discussed.

## Introduction

Imagery Rescripting (IR) is an imagery-focused treatment technique designed to alleviate involuntary and distressing images or memories and alter related beliefs and schemas. IR has been successfully applied to patients with various psychiatric disorders, such as posttraumatic stress disorder (PTBS), social anxiety disorder, obsessive compulsive disorder, major depression, bulimia nervosa, and body dysmorphic disorder (1).

To date, there have been few studies investigating the effect of IR in patients with personality disorders (PDs). One study on patients with PD found techniques of cognitive behavior therapy that focuses on childhood memories (the dominant technique being IR) to be just as effective as techniques focusing on the present (2). Furthermore, IR is considered an important component of Schema Therapy (ST), a treatment known to be successful in treating individuals with PDs (3). Most studies on the effectiveness of ST were done for patients with borderline personality disorder (BPD) (4–10).

IR in the context of ST for PDs is not primarily used to reduce intrusions, but to change the implicational meaning of schemas and childhood experiences that underlie patients' problems (11, 12). This is achieved by activating childhood memories that are assumed to underlie schemas and by helping the patient to reprocess them. The disturbing memory is imagined and when the related emotions and needs are activated, the rescripting part starts: a helping figure intervenes and changes the situation so that it has a better ending and the needs of the child are fulfilled. This helping figure can be the therapist (especially at the beginning of treatment) or another supportive person or fantasy figure. At a later stage of treatment, patients in their healthy adult mode (the part of the self that represents patients' functional parts in ST) are instructed to intervene themselves (13). During IR, patients are guided to reattribute childhood events to different causes than they did as a child, to emotionally process these distressing memories, and to experience being cared for (14).

To date, there have been only few qualitative studies on the experiences of patients with PDs with ST and its techniques. De Klerk et al. (15) interviewed patients with PDs (BPD excluded) about their experiences with ST in general, and Ten Napel-Schutz et al. (16) interviewed patients with PDs (BPD excluded) about their experiences with imagery (“safe place” and “diagnostic imagery”). There is one study that interviewed patients with BPD about their experiences with ST (17), finding that these patients experienced the experiential techniques (including IR) used during ST as confronting, but important. However, detailed information on how BPD patients perceived IR is limited. Patients with BPD tend to have multiple aversive childhood memories and are known for experiential avoidance (18) and having difficulties with relational closeness, both addressed and activated during IR. Thus, their experiences with this technique could further our understanding of hindering and facilitative factors and therefore help optimize the implementation of this powerful technique.

Therefore, the present study used thematic analysis of qualitative interviews with BPD patients receiving IR to explore the following research questions: (1) what experience do patients have with IR, (2) which factors did patients perceive as hindering and facilitative regarding IR, and (3) what are patients' perception of the effectiveness of IR.

## Method

### Recruitment and Sample

Participants in this study were recruited from the ST treatment condition of the PRO^*^BPD trial, an randomized controlled trial (RCT) comparing ST with Dialectical Behavior Therapy, conducted at the outpatient clinic of the Department of Psychiatry and Psychotherapy at Lübeck University, Germany (19). Participants aged between 18 and 65 years with a primary diagnosis of BPD were included. Exclusion criteria were a major psychotic disorder and acute severe substance dependence that necessitates detoxification treatment. More information on recruitment, diagnostics, and procedures of the PRO^*^BPD trial can be found in the study protocol (19).

Every participant included in the main study at the time of the qualitative study was approached to participate in the qualitative interview if they had had at least five months of ST, gave informed consent, and were open to participate in the qualitative interview. The total number of participants included in the analyses was 21 (18 females). The mean age of the participants was 32.76 years (SD = 10.81). The mean symptom level measured by the Borderline Personality Disorder Symptom Inventory (BPDSI) at baseline was 35.69 (SD = 8.62). The mean number of comorbid axis I and II diagnoses was 4.14 (SD = 1.98) and 0.91 (SD =.83), respectively. For demographic and clinical characteristics, see Table 1. At the time of the interview, 11 participants had already completed their treatment, one had dropped out of treatment, and nine had already received between five and 18 months of treatment (M = 11.55, SD = 4.71).

**Table 1:** Demographic and clinical characteristics of the sample (*N* = 21).

	***n***	**%**
**Gender**		
Male	3	14.3
Female	18	85.7
**Highest education level**		
Elementary school (4 years)	1	4.8
Secondary school (9 years)	1	4.8
Secondary school (10 years)	10	47.6
Secondary school (12 years)	4	19.0
Secondary school (13 years)	2	9.5
Professional school	1	4.8
University degree	2	9.5
**Employment status**		
Student	2	9.5
Homemaker	1	4.8
Honorary post	1	4.8
Employed	4	19.0
Unemployed	1	4.8
Incapacitated for work	12	57.1
**Comorbid disorders (DSM-IV)**		
**Axis I**		
Affective disorders	14	66.7
Substance disorders	6	28.6
Posttraumatic stress disorder (PTSD)	16	76.2
Other anxiety disorders (excl. PTSD)	15	71.4
Somatoform disorders	3	14.3
Eating disorders	12	57.1
**Axis II**		
Avoidant personality disorder	8	38.1
Obsessive compulsive personality disorder	7	33.3
Dependent personality disorder	1	4.8
Paranoid personality disorder	2	9.5
Histrionic personality disorder	1	4.8
Narcissistic personality disorder	2	9.5

### Procedure

Having broad experience in the research and treatment of BPD, three of the authors (EF, AA, and US) developed a semi-structured interview. The interview addressed patients' experiences with different techniques of ST. The parts of the interview relevant to the research question of this study are shown in Table 2. Interviews started with an open question on patients' general experiences with IR. Participants were free to elaborate on that open question. If they did not bring up issues during free speech, follow-up questions on specific topics were asked (see italics in Table 2). The interviewers were three graduate students in the field of psychology. None of the interviewers were involved in the treatment delivery process or had information about the study outcome at the time of the interviews.

**Table 2:** Qualitative interview on components of schema therapy—Imagery Rescripting.

Now, we are going to focus on Imagery Rescripting.
1. What are your experiences with Imagery Rescripting? • *Positive and negative experiences* • *What did you find particularly easy or difficult?* • *Costs and benefits of Imagery Rescripting*
2. What did you find helpful or less helpful about the behavior of your therapist during the performance of the technique? *If not addressed during free speech:* • *Introduction of the technique* • *Performance of the technique* • *Debriefing* • *Time management* • *Emotional support by the therapist* • Were there other factors that you found helpful or hindering regarding the performance of the technique? For example, external or internal factors, such as thoughts or emotions • Did you have the experience that Imagery Rescripting worked better or was easier on some days than on other days? Why? On which factors/circumstances was this dependent?
3. Which effects did you notice after the performance of the technique? • *If not addressed during free speech:* • *Short-term, long-term?* • *Regarding your interpersonal relationships?* • *Regarding the way you look at yourself?* • *Emotional? Cognitive? Physical? Regarding your behavior?* • *Regarding your overall life content?*
4. Is there anything important you would like to say to therapists who work with this technique in the context of schema therapy?

The interviews lasted between 52 and 126 min. Four questions were added later (see underlined items in Table 2) after interviewing the first 11 participants. These questions were assessed via telephone for six of these participants. As the other five could not be reached, analyses of these questions are based on only 16 participants. The subsequent assessment via telephone lasted 4–9 min.

All interviews were audio-recorded with a digital recorder and were transcribed using MAXQDA 2018 Standard (20). Transcription was conducted following the protocol of Dresing and Pehl (21).

All participants provided written informed consent following verbal and written explanation of the study. The research protocol and amendments have been approved by the Ethics Committee of the University of Lübeck.

### Data Analysis

Interview data were analyzed following the procedure of qualitative content analysis (22, 23). The MAXQDA software was used for data organization and precise depiction (20).

Central to the qualitative content analysis is the development of a category system. An inductive approach was taken. Data analysis began by choosing five interviews with participants with different characteristics regarding gender, age, and treatment duration. These were independently investigated by two of the authors (AR and CM), who extracted meaningful passages and attributed codes to relevant text passages. With these interviews, a preliminary category system was established. Then, selection criteria were defined using the research questions. The five interviews were processed for each of the research questions separately, by extracting relevant passages that came across as meaningful. A coding frame was developed for transparence and traceability of this process. Subsequently, the extracted passages were paraphrased, and afterwards, generalization of the paraphrases took place across all of the five interviews by keeping a low abstraction level. In the next step of the data condensation, meaningless paraphrases were deleted, paraphrases with the same meaning were summarized, and paraphrases with similar meanings were expressed by a new statement (22). Simultaneously, it was verified whether the actual statement of the patient was captured and whether the text passage was of relevance to the research question. The categories developed in this way were sorted further, summarized, or divided if necessary and brought into a hierarchical order. The process of paraphrasing, generalizing, and reducing was conducted by two authors (AR and CM) to provide objectivity and reliability and was recorded for documentation purposes. After category development, the two coders met for discussion and a consensual development of a category system. The category system was then presented and discussed in an expert group (including EF, NA, US, and DB). After adaptation of the category system based on the suggestions of the expert group, the other 16 interviews were analyzed. Afterwards, in consultation with the expert group, a final adaption of the category system was made by one of the raters (AR) in which categories were summarized, and new categories were integrated if adequate. The results are presented below. The Results section presents the phenomenological description of the data, which is closely based on the statements of the interviewed participants. The interpretation and critical reflection of patients' statements as well as conclusions for clinical practice are presented in the Discussion section.

## Results

The analysis resulted in three key domains: (1) engaging in and undergoing IR is difficult (including hindering factors), (2) there are factors that facilitate the successful implementation of IR, and (3) various effects occur after the implementation of IR. Table 3 gives an overview on the derived category system with main categories and themes.

**Table 3:** Category system.

**Domains/Themes**	***n* (%)**
**A. Engaging in and undergoing Imagery Rescripting is difficult**	
A1. Factors that hinder the initial engaging in and getting started with Imagery Rescripting	15 (71%)
A2. Factors that hinder the successful performance of Imagery Rescripting	18 (86%)
A3. Difficulties experienced after the performance of Imagery Rescripting	13 (62%)
A4. External conditions hinder Imagery Rescripting	16 (76%)
**B. There are factors that facilitate the successful implementation of Imagery Rescripting**	
B1. Facilitating structural factors	20 (95%)
B2. Therapists can be supportive in various ways	20 (95%)
B3. Internal factors facilitate engaging in and coping with Imagery Rescripting	13 (62%)
B4. The direct experience of emotions and change is the strength of Imagery Rescripting	18 (86%)
**C. Various effects occur after implementation of Imagery Rescripting**	
C1. Improved coping with emotions	8 (38%)
C2. Effect on the experience of emotions	20 (95%)
C3. Imagery Rescripting “sticks with you”	13 (62%)
C4. Understanding and questioning of internal processes	16 (76%)
C5. Change in interpersonal relationships	11 (52%)
C6. Stabilization due to Imagery Rescripting	16 (76%)
C7. Experienced effects have an influence on motivation to further engage in Imagery Rescripting	12 (57%)

### Domain A: Engaging in and Undergoing Imagery Rescripting Is Difficult

#### Theme A1: Factors That Hinder the Initial Engaging in and Getting Started With Imagery Rescripting

Fifteen participants reported experiencing problems when getting started with IR. These problems were linked to four subthemes:

##### Subtheme A1.1. Unspecific internal resistance hinders the engagement in IR

Five patients described some kind of resistance that hindered their engagement in IR without being able to specify what exactly caused this resistance: “[…] I can't access the pictures, access what, what happened, where, like a wall, what happened there, access the memory” (P8). One participant suspected a lack of acceptance of inner processes; others stated that the topic was “too painful” (P20), or that anxiety and resistance held them back: “[…] that I don't, not really, am able to really engage, because I have too much anxiety and too much resistance” (P13).

##### Subtheme A1.2. Problem with trust

Two participants stated that they had difficulties starting IR because of a lack of trust in their therapist: “[…] yes, I wouldn't try it with the new one yet, because the bond of trust isn't there yet.” (P18).

##### Subtheme A1.3. Preoccupation with daily hassles reduces the likelihood to engage in IR

Five participants reported daily hassles to be hindering factors for their engagement in IR: “[…] and to separate myself from my everyday thoughts, like, if I/you keep looking at the clock and you know ok, in half an hour, I have to get the dog and then I have to go grocery shopping real quick […]” (P10), “The more stress I have, the less likely it is that it works. Ehm, this can be emotional stress; this can be stress at work or anything else” (P20).

##### Subtheme A1.4. Specific difficulties with starting the imagery

Eleven participants mentioned specific difficulties at the start of the imagery. Two participants reported that they had difficulties talking about the distressing experience with another person: “[…] well, it cost some effort to talk about the distressing traumatic situation with someone […]” (P16). Especially, describing the situation in detail and re-experiencing it by doing so was reported to be unsettling. Four participants reported that they had difficulties with allowing and expressing emotions: “Yes, showing any emotions at all and to, at the beginning it was extremely difficult to do it” (P19). Six participants stated that they had trouble choosing an appropriate situation and imagining it: “At the beginning, it is really difficult to think of a situation on the spot” (P18). One participant reported that some important situations were too difficult to imagine. Another participant reported difficulties finding a situation when feeling low. Two participants expressed trouble to accept the technique initially, and one participant reported not to be able to take the technique seriously.

#### Theme A2: Factors That Hinder the Successful Performance of Imagery Rescripting

While in theme A1 problems with the initial engagement and getting started with IR were reported, theme A2 focuses on the difficulties that 18 participants reported while receiving IR. These difficulties were linked to eight subthemes:

##### Subtheme A2.1. Recognizing needs, verbalizing them, and accepting need satisfaction

Two participants reported having trouble recognizing and verbalizing needs during IR: “[…] I had trouble somehow, ehm, firstly to know what I would have needed and, ehm, then also to say it or receive it” (P12). Three participants expressed problems accepting support during the rescripting phase: “[…] how can I accept that now, that, ehm, I as a grown-up take my therapist back to the childhood, who then tries to fix something there” (P6).

##### Subtheme A2.2. Emotional and cognitive impediment

Some participants stated that distracting thoughts and emotions hindered the IR: “Well, hindering is, if your thoughts go in a totally different direction than they should. That happens really quickly. Takes only one word.” (P11), “Well, hindering is if sadness occurs. Sometimes anger” (P11). One participant reported having too many pictures in their mind, and another one described an emotional and cognitive confusion causing that “what the therapist actually wanted to achieve, didn't happen. The rescripting didn't work […]” (P6).

##### Subtheme A2.3. The rescripting is perceived as unrealistic

Two participants reported having difficulties because the rescripting felt unreal: “And, ehm, there is always a certain point, well I was always really aware of the fact, that it is somehow, that it is not real, that it is just a game, somehow” (P13). Three participants described the way the rescripting was done either as ridiculous, unrealistic, or exaggerated: “For me all of the therapist's attempts in this situation that I was in, seemed ridiculous, that way” (P18).

##### Subtheme A2.4. Schema modes^1^ and coping strategies getting in the way

Seven participants reported hindering modes and coping strategies during the rescripting. Two participants noticed a dysfunctional parent mode: “And if I say okay, I have to stop I can't do it any more, the parent mode is like: ‘you loser!’” (P19). Five participants reported dysfunctional coping strategies hindering them and preventing the therapist to get close to them: “Well, I there have like a wall there and can't get past it, then the therapist can do whatever she wants” (P7).

##### Subtheme A2.5. Patient and therapist drifting apart

Four participants reported that they sometimes noticed that they and their therapists were not on the same page during the IR: “Very often, ehm, it is that with/was there a new insight and was I was going in a totally different direction as, ehm, the therapist actually wanted to go” (P6). This could happen because the participants had trouble “[…] to verbalize the content that actually happens inside of you in a way that the therapist can eventually follow” (P10) or because they were distracted by new insights.

##### Subtheme A2.6. Inability to recognize and verbalize own boundaries

Two participants reported trouble recognizing, verbalizing, and paying attention to their own boundaries: “[…], that I sometimes don't recognize my boundaries and don't say ‘Okay, I am actually already beyond what I can endure’ or ‘Could we slow down a little?’” (P21).

##### Subtheme A2.7. Trouble with the therapist entering the imagery

Five participants reported having trouble trusting the therapist during the IR. Two participants had problems with the therapist entering the situation. Also, some participants reported the presence and support of the therapist during the IR as too close emotionally: “[…] especially this situation of the rescripting where the therapist, yes, like, plays with you or, ehm, I don't know, does something nice and supporting. That, ehm, it is always a little, I always feel uncomfortable at this moment. Because then it is too close. Too close emotionally […]” (P21).

##### Subtheme A2.8. Proceeding too fast during IR

Four participants reported being troubled by a fast pace both during the first implementation of IR: “I found it, I felt overrun […]”, “There was practically no explanation, but we just did it” (P21) and during subsequent IR sessions: “[…] she sometimes was too fast, yes, well, I wasn't actually ready yet” (P20).

#### Theme A3: Difficulties Experienced After the Performance of Imagery Rescripting

Thirteen patients described experiencing difficulties after the performance of IR that was linked to three subthemes:

##### Subtheme A3.1. Difficulties coping with emotional intensity directly after the intervention

Six participants reported having trouble dealing with new insights, experiences, and emotions after IR: “[…] the anger increased and, ehm, also the sadness about it and also a question mark; what do I do with it, how do I cope?” (P6). Not knowing how to deal with those led to uncertainties about guilt, to dysfunctional coping strategies, or to a feeling of shame toward the therapist. Five participants reported feeling left alone with their emotions and thoughts after the IR: “And then you got to the emotion, then it is over and then you are sitting there, that's what I mean, you're sitting there alone” (P21).

##### Subtheme A3.2. Accepting the rescripting

Four participants experienced a positive feeling right after the rescripting but reported having trouble with integrating it in the long run because they knew that the rescripting was different than the actual situation: “That just awakes needs that cannot be satisfied anymore today, and I am always asked to sugarcoat this situation […] Well, I can imagine it differently, but I know that it wasn't like that” (P9). One participant reported having trouble accepting “[…] that what I learned was just not right” (P15).

##### Subtheme A3.3. Insufficient debriefing

Seven participants reported that the IR lasted until the end of the session leading to insufficient time for debriefing: “There was the problem again that the imagery worked at the end, then the session was over and it had to be interrupted and could not be processed afterwards. And the next time I was there, it was too long ago, one week later” (P15). Without debriefing, participants kept ruminating about the situation, often felt distressed, and the need to talk about it.

#### Theme A4: External Conditions Hinder Imagery Rescripting

Sixteen participants mentioned external conditions that made it harder for them to engage in IR. These difficulties could be linked to four subthemes:

##### Subtheme A4.1. The camera prevents talking openly

Two participants reported having trouble being videotaped during IR, another one with being videotaped with closed eyes, and one participant reported not wanting to talk about specific topics in front of a camera: “External conditions was the camera at the beginning, well, that took a long time until I was ready to talk in front of a camera” (P20).

##### Subtheme A4.2. Unfavorable time frame conditions

Six participants stated that 50 min was not enough time for a session including IR: “Time is pressing. 50 minutes are over quickly. First there is the introduction, how are you? What did you do? And then doing it during the last ten minutes that is, well, you are hyped, then. That is difficult” (P2). Participants reported that they needed time in order to get access to their feelings and also to debrief after the IR. One participant reported that the time of the day of their therapy session was unfavorable: “The timing of my therapy sessions is bad, both individual and group therapy at noon, I cannot do something useful before or after, and it is during lunchtime, that is stupid.” (P13).

##### Subtheme A4.3. External noises

Twelve participants described external noises as hindering during the IR: “If there are sounds or noises from the outside. Then you can't concentrate […]” (P11). This could be noise due to a construction site, but also the ringing of a telephone or the siren of an ambulance. Some participants felt distracted by sounds, such as steps outside the therapy room or the ticking of a clock.

##### Subtheme A4.4. Lack of coziness

Three participants stated that they had wished for more coziness in order to help them settle down and feel secure: “I personally would have liked it if there somehow was, if you had the possibility to make it more comfortable somehow” (P10), “Just a blanket (laughs). If you are really exhausted and very (sighs). If you already feel totally naked emotionally, then a blanket feels pretty good.” (P5).

### Domain B: There Are Factors That Facilitate the Successful Implementation of Imagery Rescripting

#### Theme B1: Facilitating Structural Factors

Twenty participants reported structural factors that they found helpful regarding the implementation of IR. These factors could be linked to five subthemes:

##### Subtheme B1.1. Time and specific techniques for debriefing

Seven participants found it important to achieve some kind of distance to the rescripted situation before going home: “[…] enough time for debriefing at any rate, that the patient doesn't feel alone afterwards” (P18). Four participants appreciated a short conversation about what the participant's plan for the day was. One participant reported benefiting from a personal safe-place imagery that was imagined at the end of the IR. Five participants reported that it was helpful to receive or compile a written or auditory memo about the most important content of the IR to take home with them: “It was always helpful, when she wrote something down for me. Some helpful sentences or some words or/That was always very helpful. That I could take something with me” (P11).

##### Subtheme B1.2. A transparent and constant structuring is helpful

Three participants found a consistent sequence and rituals during the therapy sessions to be helpful: “[…] that we like, have our rituals about how the session goes and that this stays approximately the same. That you can rely on that” (P21). Four participants found it helpful that the IRs followed a transparent structure: “[…] there was always a clear structure, well you also knew how long it would take […] that was communicated well.” (P6).

##### Subtheme B1.3. Learning about the situation and the IR in advance

The possibility to learn about the technique in general prior to the implementation, for example, by receiving a handout, was perceived as helpful by three participants: “Ehm, yes, I found this handout actually pretty helpful. Well, to know what it is about and what is going to happen, hm” (P12). Two of them reported having appreciated talking about and choosing the situation that was to be rescripted in advance: “Mh, it was helpful if we already talked about it in the session before. Like, if I knew exactly, if I got there and knew exactly ‘ok, we will be doing a rescripting now’ […] that was nice, if you knew beforehand and could prepare yourself a little” (P15).

##### Subtheme B1.4. Therapist as an auxiliary person

Eight participants reported that they appreciated the therapist entering the imagery as an auxiliary person that made them feel supported and protected: “I did this a couple of times. And that was good. Because I suddenly, in these situations, when I felt alone and helpless, suddenly I had support. And that was a good thing” (P3), “[…] that she was there and, ehm, protected me and, ehm, told my parents that you can't treat children like that and such” (P13). Also, the therapist providing different advice and ideas was perceived as helpful.

##### Subtheme B1.5. Gradually increasing the difficulty

Four participants stated that they appreciated starting IR with easier situations: “[…] well, that was very helpful, that you started slowly and not with the hard ones” (P14). One participant reported being very anxious about IR, and a slow start with a stepwise increase of the difficulty reduced this anxiety: “Well, I was always afraid of, of these exercises […] she led me cautiously, starting with easier situations and then the situations became more difficult, like that” (P19).

#### Theme B2: Therapists Can Be Supportive in Various Ways

Twenty participants mentioned that certain therapist-related factors helped them to engage in IR. These factors were linked to three subthemes:

##### Subtheme B2.1. Adjusting to the needs of the patient

Twelve participants appreciated the flexibility of their therapists regarding their needs: “I found it especially helpful that my therapist was very flexible” (P10). Eight participants reported that the therapists were paying attention and adjusting to their needs carefully while introducing IR: “[…] She gave me silence and time. Showed me a breathing technique. And told me ‘if you are ready, let me know’” (P7). Some participants needed a slow introduction with breaks; others appreciated a quick start.

##### Subtheme B2.2. Therapist provides safety and contact

Four participants reported that they appreciated knowing they could stop the IR at any time: “[…] well, I found it helpful, ehm, that my therapist said, that I could say stop at any time, if it becomes too much” (P12). Six participants mentioned that it was helpful to know that they could get support if necessary: “[…] I knew, that I can come by or whatever/well, I found this helpful, somehow” (P16). Participants found it reassuring to know that they could call or come by the clinic at any time or leave a message, even if they would not reach their therapist in person.

##### Subtheme B2.3. Supportive competences of the therapist

Thirteen participants reported that they felt they were in good hands with their therapist. Participants described their therapist as authentically expressing understanding, care, and compassion: “What he did really well was to express this empathy and, ehm, well, you could take it seriously, if he said ‘I understand how you feel’ and stuff […]” (P15). The therapists managed to express closeness, while, at the same time, keeping an adequate distance: “And, ehm, she manages exactly to keep the distance, that is a great skill, ehm, not to come too close but also not to be too far away. Ehm, and that works for me, I am absolutely happy there.” (P20). Three participants found it helpful that their therapist captured the essential topics quickly: “No, I think she is really good at it because she manages to[…] capture the essentials, always, ALWAYS get the principal point, that is what it's is about” (P20). Also, the therapist remembering details of the rescripted situations was appreciated by the participants.

#### Theme B3: Internal Factors Facilitate Engaging in and Coping With Imagery Rescripting

Thirteen participants stated that internal factors helped them to engage in and cope with IR. These internal factors were linked to two subthemes:

##### Subtheme B3.1. Access to emotions facilitates IR

Six participants experienced getting access to and feeling their emotions as helpful during the IR: “Also, with the emotions you dwell on that a little bit, because you know, oh, that gets to you. That now it gets down to business. But the thing is, this is healing, and if you let it get close to you” (P1), “Ehm, yes well when I just, ehm, could engage more, well, so emotionally and really be more in the feeling and in the situation, than it worked better somehow.” (P12).

##### Subtheme B3.2. A balanced, positive state of mind facilitates coping with IR

Nine participants mentioned that the IR worked better, and that they were better able to cope if they were in a good mood and feeling better in general: “Well, if I felt better in general and had a good day, then it was easier” (P17), “Ehm, I have to be pretty, yes say, in a good mood, that I am not totally down the drain then, ehm, because it takes a lot of strength.” (P21).

#### Theme B4: The Direct Experience of Emotions and Change Is the Strength of Imagery Rescripting

Eighteen participants highly appreciated the possibility of experiencing emotions directly through IR. But even when the IR did not work, two patients reported having benefitted from the technique. Participants' statements on this topic could be linked to two subthemes:

##### Subtheme B4.1. Learning about dysfunctional coping mechanisms

Two participants reported that they did not perceive it as a setback if the IR somehow did not work, but by working out which coping mechanisms prevented the successful implementation, this led to new insights: “[…] therefore it is actually a two-way model which in the end leads to the same goal. And it doesn't matter if I go left or right. Ehm, the result is correct and that, THAT you can feel clearly and I think that is really important, hmm.,” “[…] if I wasn't able to get into it or let it happen, ehm, you can conclude clearly, that the brain is working against it, somehow. Ehm, and if you approach the situation like this, you can get there another way, maybe not as close as if it works, but pretty close anyway” (P20).

##### Subtheme B4.2. The emotional experience as a big advantage of IR

Fifteen participants reported that they perceived it as an advantage that IR passes by the cognitive level and goes directly to the emotional level, and that it lets the participant experience the situation intensely: “Yes for me it had a bigger effect, if I really imagined it. And really enter it, also in the emotion. Instead of just talking about it” (P17). One participant described not having captured the real problem until taking the role of the child. Another advantage of IR that was mentioned by five participants was the instant experience of change when they experienced that someone stood up for them and protected them during the IR: “Well, you have this instant success, say, together with the therapist and, ehm, you can instantly change something. But you change it yourself, well, I mean, ehm, as long as you don't imagine it, the therapist can talk as much as he like […]” (P21). Furthermore, participants appreciated that they had the possibility to actively change distressing feelings during IR.

### Domain C: Various Effects Occur After Implementation of Imagery Rescripting

#### Theme C1: Improved Coping With Emotion

Eight participants mentioned improvements of their emotion regulation skills. These improvements were linked to two subthemes:

##### Subtheme C1.1. Being able to allow emotions

Four participants stated that IR made it easier for them to accept and allow emotions: “[…] I can, ehm, better accept now, yeah, that, ehm, that I can be sad at times.” (P20), “[…] well, regarding emotions, that I allow them and that I had that experience, then, that I got better at this” (P8).

##### Subtheme C1.2. Coping with emotions appropriately

Seven participants reported being able to cope with emotions more appropriately. Three participants could express and verbalize their feelings better: “[…] that I for some part somehow verbalize emotions and say ‘Somehow, I think this is sad right now or makes me mad’ or something” (P10). Some participants described recognizing emotion and reacting to them more appropriately: “Ehm, or I can counteract and I say ‘ok, now this happens and such and so, now you have to do this and that and that’ yes” (P20). One participant reported that having better control over emotions led to a reduction in self-injury and increased their ability to ask for help and support.

#### Theme C2: Effect on the Experience of Emotions

Twenty participants reported that IR had an effect on their emotional state. These effects were linked to two subthemes:

##### Subtheme C2.1. Overwhelming emotions

Seventeen participants reported experiencing adverse emotions during the IR: “It could end in crying fits, in fits of anger, ehm, well in intense emotions” (P18). Participants reported that they often felt worse initially after a session with IR, which a lot of participants found difficult and strenuous: “[…] that it really, ehm, overwhelms you, by falling back into this situation” (P21).

##### Subtheme C2.2. More balanced internally

Twelve participants reported long-term benefits including feeling more relaxed, serene, balanced, satisfied, positive, secure, and calm as well as less impulsive and anxious. Especially the reduction of tension and the feeling of calmness were emphasized by 10 participants: “That I was more relaxed, physically as well as mentally, let's say. The tension was almost gone completely and I could let go emotionally” (P8).

#### Theme C3: Imagery Rescripting “Sticks With You”

Thirteen participants described that the IR lingered during the following days. Participants' statements were linked to two subthemes:

##### Subtheme C3.1. Exhaustion

Ten participants reported a great exhaustion after the IR: “Ehm, (…) and then I felt really exhausted and totally totally weak like smashed to the ground and that like went away during the day” (P20). Participants reported a physical exhaustion as if they were “physically overstrained” (P17): “Like I was running a marathon” (P17). This was described as a short-term effect.

##### Subtheme C3.2. Enhanced cognitive engagement

Another effect of IR described by eight participants was repetitive thoughts or rumination about the rescripted situation: “That you really couldn't think about anything else afterwards. Your thoughts were constantly with this scene” (P2). This mental occupation could last up to several days: “Ehm, yes it's regularly been on my mind al lot for some time. Well, yes sometimes only a couple of days or so and then I could put it aside pretty well, like, sometimes also longer” (P12).

#### Theme C4: Understanding and Questioning of Internal Processes

Sixteen participants mentioned that IR led to a broader understanding of their schemas, problems and needs. Participants' statements were linked to three subthemes:

##### Subtheme C4.1. Recognizing schemas and modes better and weighing up whether acting on them is reasonable

Five participants stated that they could recognize schemas better and were more aware of them: “I notice it soo/sooner now, so that I can say, okay, it's just not going so well right now and, ehm, but then I also know, that it is just, which part this is, like, and that it is not necessarily all of it, so that's pretty good in the long run” (P10). One participant noticed that, by being more aware of schemas, it was possible to actively decide whether acting according to it was reasonable or not: “Ehm, well, I noticed, well, with the schema somehow or was more aware of the schemas and, ehm, yes, I then knew that it is such a schema and I can pay closer attention to whether it is reasonable to act on it or not” (P12).

##### Subtheme C4.2. Understanding of the origin and connections of problems

Eleven participants reported being able to understand their problems and the origin of their problems better: “That I now understand, well that I better understood the origin of my problems better, because of the imagery.” (P7). One participant described it like puzzle pieces forming into a picture due to IR. Two participants stated that they realized that “[…] not everything that happened was right. That there are definitely things that you have learned or that were instilled in you that, ehm, can be clearly wrong” (P15). One participant reported that understanding their reaction to specific situations led to acting differently in these situations. Two participants reported that due to IR, they understood the origin of their, sometimes unsuitable, feelings.

##### Subtheme C4.3. Recognizing and satisfying past and present needs

*Eight* patients observed that, due to IR, it was easier for them to become aware of and/or to take care of their needs: “Well, and with some topics I say ‘stop, please stop, because otherwise something will happen’. So, I'm more aware of it. I wasn't able to before, then I would go into dissociation and it would be over.” (P19). Two participants stated that they were better able to recognize and verbalize the needs of the child during the IR. Four participants were also able to recognize their needs and boundaries in present situations. Seven participants reported also to stand up for their needs by setting boundaries or leaving unfavorable situations as well as by asking for help or verbalizing problems: “I got more courageous by saying something about it, ehm, which wouldn't have been said otherwise. And this is also nice” (P3).

#### Theme C5: Change in Interpersonal Relationships

Improvements in interpersonal relationships were experienced by eleven participants and were connected to three subthemes:

##### Subtheme C5.1. Improvement of family life

Three participants reported noticing an improvement in their family life. One participant experienced an improvement in the relationship with their parents, and another reported being able to forgive a family member and therefore feeling better toward this person. The third participant noticed a reduction in arguments, an improvement in the relationship with family members, and the ability to allow closeness: “[…] well, to accept closeness, especially from my family, those are the closest people, that improved dramatically” (P20).

##### Subtheme C5.2. Greater openness to others

Five participants reported a change regarding their attitude and behavior toward other people: “And I'm better with people” (P11). Participants reported being more open, friendly, and relaxed as well as less mistrusting and anxious toward others. One participant reported this change being also noticeable to others: “[…] I am told I am somehow more open and […] also, ehm, talked to some people about the situation, well, which I never did before, yes” (P16).

##### Subtheme C5.3. Intensifying of interpersonal conflicts in dysfunctional relationships

Three participants reported that some relationships worsened due to IR: “Distance from my mother. Even a little further and that I let her words get to me less, yes that” (P18). This concerned especially dysfunctional relationships. These participants described an intensifying of anger toward and accusations against specific persons.

#### Theme C6: Stabilization Due to Imagery Rescripting

Sixteen patients observed that they felt better in general, more stable, and that their quality of life had improved due to IR. Participants' statements were linked to six subthemes:

##### Subtheme C6.1. Understanding oneself better and being more forgiving of oneself

Eight participants reported noticing positive changes in their self-perception. They were more forgiving of themselves and could accept themselves better: “Yes well, I became more understanding and I can put a lot of things in context more. Condemn myself less” (P17).

##### Subtheme C6.2. Treating oneself better

Six participants mentioned treating themselves and their bodies better: “Like, I have a little more respect of my body” (P10). They reported eating enough without binging as well as reducing self-injury and excessive exercise. Also, participants stated that they were less self-blaming and self-critical: “Well, before it was like, that I, for as far as I am concerned, put more blame, blame myself, where maybe there is none at all, and, ehm, afterwards it was obvious ‘no, I'm not to blame, maybe it wasn't my fault’” (P8).

##### Subtheme C6.3. “Relief and liberation”

Eight participants stated that they experienced relief right after the IR: “Ehm, the internal knot was loosened a little. Because I have such a restlessness and such a knot inside me. Ehm, this was loosened a little regarding this at that moment. Sensed relief.” (P7). Three participants explained this sense of relief with the fact that they had addressed the distressing situations, thoughts, and emotions for the first time: “Positive experience was, that there was, afterwards, usually an actual feeling of relief quite instantly […] the ghost of the past was gone a bit, so to say” (P10).

##### Subtheme C6.4. Improved coping with difficult situations

Three participants stated that they could better cope with their past and have closure: “Ehm, and that I could retrospectively store the past experiences differently, ehm, emotionally. And cope with the bad things from the past differently” (P14). One participant mentioned to be better able to cope with schema modes. Three participants stated that they would deal better with situations they had trouble with in the past: “Well, even after the first application that I had then, that let's say got more to the bottom of it, I could deal with such situations better” (P8).

##### Subtheme C6.5. Improvement of physical wellbeing

Two participants reported a reduction of physical symptoms, such as dizziness, palpitations, and pain: “Physically, ehm, I don't have like, like imagined gouts any more. Well, I went to my physician pretty often and then this hurt and that hurt and I don't need that any more” (P20). One participant also reported noticing an improvement regarding concentration and attention.

##### Subtheme C6.6. Stabilization due to the experience of support

Four participants mentioned the experience of not being alone, of getting help, being safe, and being loved to have helped them stabilize: “I benefitted from it in so far as that I for the first time had the feeling, I get support. Yes, just to have this feeling: I am supported, I am not left alone the way I am used to. That was great. Well, I definitely got something out of that” (P3).

#### Theme C7: Experienced Effects Have an Influence on the Motivation to Further Engage in Imagery Rescripting

Twelve participants noticed an influence on their motivation to continue working with IR. Participants' statements were linked to two subthemes:

##### Subtheme C7.1. Knowing about the distressing effect reduces the motivation

Two participants reported that they sometimes were unenthusiastic about the technique if they did not feel well that day. Two participants stated that they did not like the technique because it would get very emotional: “That I don't like it (laughs), because it just goes into the emotional level a lot” (P8). Two participants mentioned that they did not appreciate the technique and would not want to repeat it because they would feel especially bad afterwards: “Yes, because why? I don't see any sense in it. Well, that has just only brought up sadness somehow” (P9).

##### Subtheme C7.2. Knowing about effectiveness enhances the motivation

Six participants reported that they initially found the technique strenuous and painful but would repeat it at any time again because of the various, partially immediate positive effects: “Ehm, in the long run it was great. Ehm, because there is noticeably a change, ehm, well a change. And I also think that any following imageries will be even more helpful, ehm, yes, in the long run I look at it all in a positive way.” (P14).

When asked to weigh the costs and benefits of IR, four out of 21 participants (19%) estimated the costs of IR to be higher than the benefits, three (14%) thought that the costs and benefits were balanced, and 14 (67%) estimated the benefits to be higher than the costs.

## Discussion

The aim of this study was to explore the experiences of patients with BPD with the technique of IR, to examine which factors patients perceived as helpful or hindering when receiving IR, and what effects on their everyday life were observed after receiving IR. A thematic analysis of participants' statements revealed that patients found engaging in and undergoing IR difficult and named several hindering factors. Furthermore, they also reported several factors that facilitate the successful implementation of IR and experienced various effects after the implementation of IR.

### Difficulties and Hindering Factors

The majority of the participants reported factors that hindered the successful implementation of IR. Hindering external factors included stress, noise, a sterile environment of the therapy room, and the session length being too short for IR, leaving little time for debriefing. This feeling of time pressure is in line with findings of de Klerk et al. (15).

Regarding hindering internal factors, participants mentioned having trouble entering the imagery, a lack of trust, or getting access to and verbalizing emotions and needs. Some had trouble finding or choosing a situation, a problem which was also described by Arntz and Weertman (11). Some participants mentioned difficulties in accepting the rescripting, depreciating it as unrealistic or ridiculous. This could indicate resistance or dysfunctional coping strategies standing in the way of the patients engaging in IR. Interestingly, this was mainly the case with participants who estimated the costs of IR to be higher than the benefits. Arntz and Weertman (11) also described this problem and labeled it “resistance” to the technique. Adequate information on IR and addressing these issues early in therapy could promote the successful implementation of IR.

### Facilitating Factors

Facilitating external factors included adequate debriefing, the use of the therapist as auxiliary person, and the possibility of stepwise increasing the level of difficulty. Ten Napel-Schutz et al. (16) found that patients feel insecure and scared when they were not informed about the duration and course of the technique of IR. Participants in our study also valued a clear and consistent structure as well as information about the technique and preparation of the IR beforehand. Facilitating factors regarding the behavior of the therapist included the therapist being able to capture and remember central points as well as the flexibility of the therapist in adjusting to the needs of the individual patient. Also, giving the participants a sense of control by informing them about the possibility to interrupt the exercise and contact the therapist if necessary as well as providing safety and reliability by building a reliable therapeutic relationship were mentioned as helpful factors. This is in line with earlier findings in patients with other PDs receiving ST that reported the supportive therapeutic relationship and the reachability of the therapist to be helpful (15). In their meta-synthesis, Katsakou and Pistrang (24) analyzed qualitative studies on BPD patients receiving various treatment methods other than ST. They identified helpful factors that were similar to those reported in our study, specifically regarding the therapist providing safety and being understanding and supportive as well as flexible and approachable.

### Effects of Imagery Rescripting

Participants mentioned various effects that occurred after the implementation of IR in the context of ST. Participants reported initial unpleasant effects to be high emotionality, exhaustion, and enhanced preoccupation with the targeted memory. Long-term effects were mainly positive and included a better understanding of the origins and interrelations of their problems and schemas as well as improvements of emotion regulation skills and a better understanding and verbalizing of their needs. Also, participants reported improvements regarding interpersonal relationships. Furthermore, participants described themselves as more stable, and some participants expressed relief. Other effects included an improvement in problem solving skills and physical well-being. In the study by Tan et al. (17), patients with BPD receiving ST also reported broad therapeutic gains in various areas of life, while perceiving IR and other experiential techniques as emotionally confronting. The majority of the participants in our trial estimated the benefits of IR to be higher than the costs. This is in line with the study by Ten Napel-Schutz et al. (16), who found that the participants on the one hand experienced imagery as confrontational and stressful but on the other hand appreciated its value with regard to recognizing schemas, the understanding of their problems as well as emotion regulation. Interestingly, the areas of change reported by patients with BPD receiving other treatment methods than ST seem to be similar to those reported by the participants in our study, specifically regarding self-acceptance, new ways of relating to others, and taking control of emotions and thoughts (24, 25).

## Limitations and Strengths

To our knowledge, this is the first qualitative study specifically exploring BPD patients' experiences with IR. However, participants interviewed in this study received IR in the scope of ST in the same outpatient clinic. For this reason, generalizability is limited, and including patients with BPD receiving IR from other institutions is warranted. Also, in this study, more female than male participants were interviewed, and only one participant who dropped out of treatment could be motivated to participate in the qualitative interview. It should also be considered that due to the setting at a university clinic, participants in this study were complex BPD patients with high BPD symptom severity and comorbidity. Particularly, almost 40% of the participants suffered from a comorbid avoidant PD, which is likely to be associated with high experiential avoidance. These patients may show a higher resistance to such an emotionally intensive technique.

Regarding the effects of IR reported in this study, it is important to keep in mind that participants received IR in the context of ST. Therefore, although the reported effects, such as improvements in interpersonal relationships and emotion regulation, were linked to IR by the participants, they could also be related to the combination of ST techniques the participants received. On the other hand, IR is a technique that might lead to an implicit change; therefore, it is possible that participants may not have linked important changes to IR. Generally, it is difficult to attribute specific effects to specific techniques delivered in a complex treatment program.

As inclusion was based on convenience sampling and the recruitment of participants was challenging because of limited reachability and willingness to participate, selection bias is likely to have occurred. As the participants who dropped out of the treatment might have been less likely to answer the telephone and take part in the interview, it is also possible that participants who did not benefit from or those who were discontent with ST techniques were less likely to be interviewed.

For this publication, the example quotes for the subthemes were translated into English, which might have caused some loss of meaning.

A strength of this study was the consensual analysis of 25% of the interview material by two raters who were not involved in the treatment process, as well as the discussion of the category system within an expert group.

## Clinical and Research Implications of the Findings

Because of the subjective nature of qualitative findings, the present study can only raise tentative suggestions for clinical implications and hypotheses for testing in further research.

### Clinical Implications

Based on the statements of the participants of this study, therapists can keep in mind several points to optimize the frame conditions for IR when working with patients with BPD:

#### Information About Imagery Rescripting, Structure, and Transparency

Providing the patients with adequate information and preparation before the implementation of IR and following a clear structure and routine during the IR can help avoid feelings of insecurity. Also, a clear explanation of the rationale of the technique and its possible effects can enhance the commitment of patients; a handout for the patient to read as homework assignment and addressing the patient's questions during the next session is useful. However, extensive preparation and exploration might also have disadvantages for some patients as it might enhance anticipatory anxiety and avoidance. Indeed, in our study, some participants appreciated a quick start, whereas others preferred a slow introduction with a stepping up of the difficulty. Therefore, after the introduction of IR, a quick start with a first IR on an easily tolerable memory might help overcome anxiety and avoidance and lead to a positive first experience with IR. It is also important for therapists to explain that it is not possible to change what happened or to erase the memory, but that IR aims to change the meaning of adverse childhood events. For this reason, a different ending is imagined in which the perpetrator is stopped and the child's needs are fulfilled. This ending does not have to be realistic; it can also be fantastical.

#### Reducing distraction and stress

Therapists can try to minimize noise, or they can ask whether the patient is distracted by sounds. Therapists can try to reduce day-to-day stress by helping the patient to settle in (for example, with the support of a relaxation exercise) before starting the IR. They can also advise patients to come in early and do a relaxation exercise in the waiting room by themselves.

#### Optimizing Time Frame Conditions and Closure of the Session

Therapists can reduce time pressure by starting promptly with the IR or by scheduling longer sessions. For example, for treatments for PTSD with IR, the session length is up to 90 min to allow for enough time for emotional processing and debriefing (26). Also, therapists can allow for enough time for the patient to process each step during the IR and leave enough time for debriefing and stabilization after the IR. As a rule of thumb, we would suggest to leave at least 15 min at the end of the session for debriefing. Also, therapists can use specific techniques for closure, such as helping the patient to plan helpful activities after the session, closing the session with a safe place imagery or with a written schema memo or an audio-flashcard summarizing the most important content.

#### Providing Safety

Agreeing to use a stop sign can give patients a sense of control and safety. A feeling of safety can be increased further by providing comfort, for example, by using blankets or by moving into a corner of the room. Also, paying close attention to the patients' reactions and staying in constant contact with them, checking at each step of the way what they experience, can prevent a drifting apart of the therapist and the patient and can help them to verbalize emotions, needs, and boundaries. By paying close attention to the patients' reactions and by debriefing elaborately, hindering modes and coping strategies can possibly be recognized and dealt with in the early stages of treatment.

#### Dealing With High Emotionality

Besides the suggestions mentioned above, we would recommend that therapists inform patients that IR may evoke a wide range of feelings, and that it can lead to emotional pain and distress. This may trigger coping modes to ease that pain. However, therapists should explain that all these emotions are natural reactions that only lead to problems when they are suppressed. Having these emotions is not a sign that the treatment is not working, but that it is a natural part of the recovery process. Therapists should also assure the patients that they will help them to deal with these emotions. They should keep in mind the patients' “window of tolerance,” the range of optimal arousal states in which emotions can be experienced as tolerable and experiences can be integrated (27). Therapists may help patients to persevere with the technique and to reduce emotional distress by praising the patient for engaging, framing the emotions the patient experiences as welcome and important and by validating those emotions. Also, therapists can express compassion for the emotional distress and exhaustion and remind the patients of the reasons why this is important.

### Suggestions for Further Research

Interestingly, only a few participants mentioned that a lack of trust in the therapist was a problem, although this patient population is known to have trust issues. This could be the case because all of the participants included in this study had already had five months of ST, and a reliable therapeutic relationship had already been established. Moreover, after five months of treatment, participants may have already shown a reduction in experiential avoidance and coping strategies that may have facilitated the performance of IR. Future research investigating IR as a stand-alone treatment for patients with BPD or IR implemented during the early stage of therapy could shed light on the question whether a stabilization phase is necessary to establish a reliable therapeutic relationship and to overcome experiential avoidance. Such a design would also help to identify effects that are directly linked to IR. Some participants in this study reported that knowing about the initially distressing emotions associated with IR would reduce their motivation to further engage in IR. Future research could investigate if there is a relationship with attrition rate or with a reduced application of IR and on how client individual differences can be taken into account in assessing how conditions for IR can be optimized.

Some participants evaluated the costs of IR to be higher than the benefits. There was no indication that this was due to an earlier treatment stage or a lower frequency of IR exercises. Interestingly, these participants were more likely to state that IR felt unrealistic, or that they had trouble accepting the rescripting. Those participants only reported few positive effects of IR, and all but one stated that the distress experienced during IR led to a reduction in their motivation to continue IR exercises. Therefore, investigating discontent patients and drop-outs more thoroughly could help to identify and prevent hindering mechanisms.

## Conclusion

Patients with BPD experience IR as highly emotional and exhausting. Preparation, a consistent structure, and transparent communication, as well as an adequate debriefing and being particularly attentive to the patients' needs, can help to recognize difficulties early on and to satisfy the patients' need for safety and control. Hindering and facilitating factors that were identified in this study can help therapists to optimize the implementation of IR. If implemented successfully, IR can be a powerful technique that is valued by patients and can help to generate improvement in various areas of the patients' life.

## Data Availability Statement

The datasets presented in this article are not readily available because only individual patient data which do not allow any conclusion on patients' identity will be shared with researchers who provide a methodologically sound proposal. Requests to access the datasets should be directed to anja.schaich@uksh.de.

## Ethics Statement

The studies involving human participants were reviewed and approved by the ethics committee of Lübeck University, reference number 13-005. The patients/participants provided their written informed consent to participate in this study.

## Author Contributions

AS: drafting main body of the manuscript, revising manuscript following feedback, involved in organization of logistics, data management, and recruitment of patients. DB: qualitative research counseling, involved in conception and design of the qualitative study, training of interviewers and coders in qualitative data assessment, and provided critical revision of the manuscript. AR: recruitment of participants, conduction of interviews, transcription, coding, and provided critical revision of the manuscript. CM: coding and provided critical revision of the manuscript. NA: involved in organization of logistics, data management, recruitment of patients, and provided critical revision of the manuscript. SK: involved in organization of logistics, data management and recruitment of patients, and provided critical revision of the manuscript. AA: external ST and IR expert, involved in the development of study protocol and development of the qualitative interview, and provided critical revision of the manuscript. US: involved in conception and design of the study, development of the study protocol, development of the qualitative interview, and provided critical revision of the manuscript. EF: coordinating investigator, initial conception and design of the study, development of the study protocol, development of the qualitative interview, implementation of the two treatment programs, and supervision. All authors read and approved the final manuscript.

## Conflict of Interest

EF, AA, and US have provided trainings and/or published books on ST and IR. The remaining authors declare that the research was conducted in the absence of any commercial or financial relationships that could be construed as a potential conflict of interest.

## Footnotes

^1^Participants refer to schema modes of their mode model. In ST, a schema mode refers to the schemas, coping responses, and behaviors that are currently active for an individual. Schema modes can be triggered by emotional events, and patients with PD tend to shift frequently between modes.
